# Novel Picornavirus in Lambs with Severe Encephalomyelitis

**DOI:** 10.3201/eid2505.181573

**Published:** 2019-05

**Authors:** Leonie F. Forth, Sandra F.E. Scholes, Patricia A. Pesavento, Kenneth Jackson, Adrienne Mackintosh, Amanda Carson, Fiona Howie, Kore Schlottau, Kerstin Wernike, Anne Pohlmann, Dirk Höper, Martin Beer

**Affiliations:** Friedrich-Loeffler-Institut, Greifswald-Insel Riems, Germany (L.F. Forth, K. Schlottau, K. Wernike, A. Pohlmann, D. Höper, M. Beer);; SRUC Veterinary Services, Edinburgh, Scotland, UK (S.F.E. Scholes, F. Howie);; University of California–Davis, Davis, California, USA (P.A. Pesavento, K. Jackson);; APHA Veterinary Investigation Centre, Carmarthen, Wales, UK (A. Mackintosh);; APHA Veterinary Investigation Centre, Penrith, UK (A. Carson)

**Keywords:** encephalomyelitis, encephalitis, novel, ovine picornavirus, disease, sheep, lambs, neurologic, viruses, United Kingdom, England, Scotland, Wales

## Abstract

Using metagenomic analysis, we identified a novel picornavirus in young preweaned lambs with neurologic signs associated with severe nonsuppurative encephalitis and sensory ganglionitis in 2016 and 2017 in the United Kingdom. In situ hybridization demonstrated intralesional neuronotropism of this virus, which was also detected in archived samples of similarly affected lambs (1998–2014).

In 2016 in Scotland, and in 2017 in Wales, progressive neurologic signs were observed in young lambs. These cases were associated with nonsuppurative encephalomyelitis predominantly involving gray matter, including neuronal necrosis/neuronophagia and ganglionitis consistent with lesions caused by a neuronotropic viral infection. The target sites included cerebellar roof nuclei in the Purkinje molecular layer of the cerebellum, caudal brainstem nuclei (red nuclei and vestibular complex), and all levels of spinal cord examined (cervical, thoracic, and lumbar segments) located mainly in the ventral horns and dorsal root ganglia (Figure 1, panels A, B). Severe lesions were found consistently in the spinal cord. Louping ill virus, a common etiologic agent of nonsuppurative encephalitis in ruminants in Great Britain, was ruled out on the basis of the clinicopathological presentations and neuropathology, as well as serology. The observed lesion distribution and age of affected sheep were inconsistent with other possible neurotropic viral infections, such as Borna disease virus (*1*).

**Figure 1:**
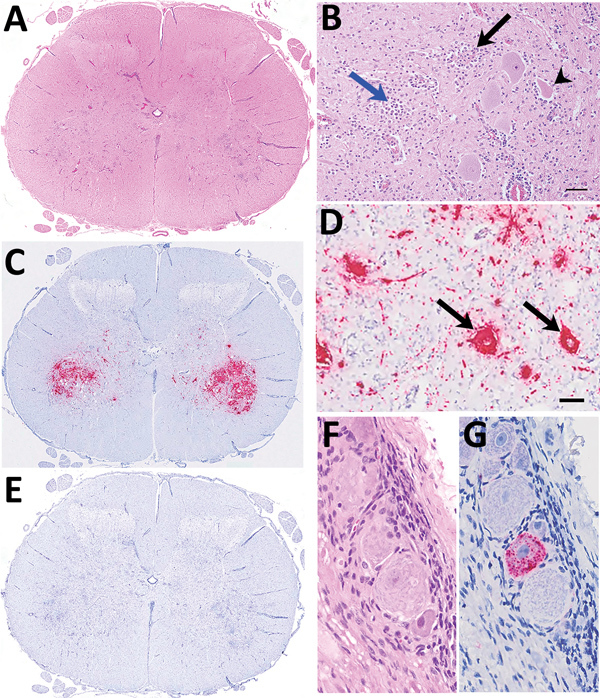
Histologic and in situ hybridization findings in cervical segment 7 of the spinal cord of 3-week-old lamb in Scotland in study of picornavirus in lambs with severe encephalomyelitis. A) Tranverse segmental view with hematoxylin and eosin stain under low power. B) Hematoxylin and eosin stain under high power. Nonsuppurative myelitis is oriented on the ventral horn involving neuronal degeneration with satellitosis (black arrow), neuronophagia (arrowhead), and glial nodule formation (blue arrow), accompanied by perivascular mononuclear cell accumulation and gliosis. Scale bar indicates 60 μm. C) By in situ hybridization, ovine picornavirus (OvPV) probe hybridization (red) predominates in the region of ventral horn poliomyelitis. D) Viral probe hybridization is dense in neuronal cytoplasm (black arrows) and within presumed neuronal extensions within the neuropil. Scale bar indicates 60 μm. E) Using an unrelated probe, no hybridization is detectable. F, G) Probe hybridization within scattered individual neurons within a spinal ganglion (F, hematoxylin and eosin stain; G, OvPV probe in situ hybridization). Original magnification ×400.

## The Study

In 2016, we subjected a sample from an affected lamb’s cerebrum to metagenomic analysis (Appendix) with read classification using RIEMS (*2*). From the resulting dataset (2 million reads), 99.97% were classified, but only 1 read was reliably classified as a viral sequence. This 326-bp read showed the highest sequence identity with the 5′ untranslated region (UTR) of the genome of human rhinovirus (81.3%), belonging to the family *Picornaviridae*, genus *Enterovirus*. For further validation and sample screening, this viral read was used to design a quantitative reverse transcription PCR (qRT-PCR) assay (Appendix). Using this qRT-PCR, we analyzed lambs that, in 2017, received diagnoses of nonsuppurative encephalomyelitis and ganglionitis typical of neuronotropic viral infection; all 3 animals tested positive (Table 1). We detected the highest viral loads in the cerebellum and spinal cord (PCR quantification cycle [C_q_] 18–21), where the most severe lesions were also observed. We detected lower loads in the cerebrum (C_q_ 30), ileum (C_q_ 29–32), tonsil (C_q_ 32–36), and mesenteric lymph node (C_q_ 34–36). Lung and spleen samples were negative, as were CNS samples from 2 additional lambs from the Scotland flock with compressive spinal cord lesions and no evidence of nonsuppurative encephalitis (Table 1).

**Table 1:** Investigations of neurologic signs in lambs in 2016 and 2017 in Scotland and Wales *

Year	Flock	Case information	Case no.	EM	Tested tissue	C_q_	OvPV reads, no. (%)†
2016	I	Progressive neurologic signs including tetraparesis, recumbency, ataxia, and death were observed in several lambs beginning at 2–3 weeks of age in a flock of 200 ewes that was accredited free of maedi-visna virus. The lamb submitted for investigation (S012722-2) was being artificially reared. Additional lambs showed similar clinical signs, but they recovered apart from residual slight paresis in one limb.	S012722–1	–	Cerebrum	NA	0
			S012722-2	+	Cerebrum	29.2	1 (0.00005)
2017	I	Neurologic signs resulting from spinal cord compression by a vertebral abscess.	S014148	–	Cerebrum	NA	NP
					Spinal cord	NA	NP
2017	II	Approximately one third of a group of 60 young pet lambs (mainly orphan lambs or triplet lambs because of insufficient milk to rear 3 lambs) being artificially reared were affected in a flock of 650 ewes. Clinical signs reported were stiff back legs, tremors that became exaggerated on handling, progressing to lateral recumbency, terminal seizures, and death or euthanasia. One lamb with mild clinical signs recovered.	S014175	+	Cerebrum	28.1	NP
					Spinal cord	18.2	1,383 (0.06)
			S014176	+	Cerebrum	26.1	NP
					Spinal cord	27.6	NP
			S014177	+	Cerebrum	27.9	NP
					Spinal cord	19.7	590 (0.02)

*Testing by quantitative reverse transcription PCR for ovine picornavirus and by metagenomics. C_q_, PCR cycle quantification; EM, encephalomyelitis; NA, not applicable (C_q_ value in RT-qPCR >45); NP, not performed; OvPV, ovine picornavirus; +, confirmed nonsuppurative encephalomyelitis and ganglionitis; –, no evidence of nonsuppurative encephalomyelitis and ganglionitis; instead, compressive lesions in the spinal cord. † Number of OvPV reads in the metagenomics dataset, total (proportion).

We subjected spinal cord samples from selected lambs affected in the 2017 lambing season to metagenomic analysis (Appendix), which yielded datasets with 2.4 million reads each. RIEMS analysis classified 99.9% of the reads, with 900 and 406 reads as sequences related to *Picornaviridae* (genera *Enterovirus* and *Sapelovirus*), and several unclassified species with relatively low sequence identities (64.8%–96.8%). Several reads were classified only after translation into amino acid sequences and comparison to the protein database. We found no evidence of other pathogens that could have resulted in the neuropathological manifestations.

**Figure 2:**
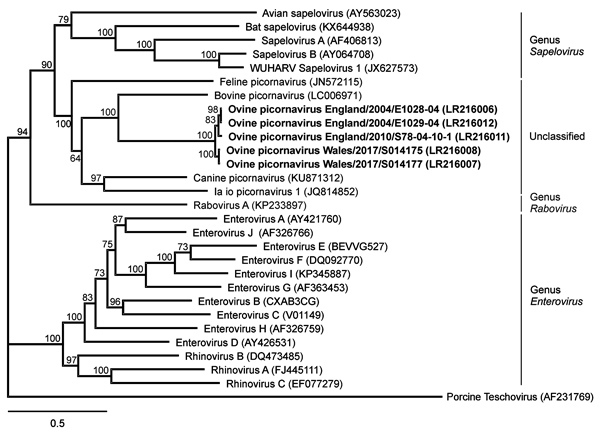
Phylogenetic relation of ovine picornavirus to other picornaviruses of the genera *Sapelovirus*, *Rabovirus*, and *Enterovirus*, as well as unclassified picornaviruses. The maximum-likelihood phylogenetic tree is based on complete coding sequences and calculated by IQ-TREE version 1.6.5 (http://www.iqtree.org) with the best-fit general time reversible plus empirical base frequencies plus free rate model 5. Teschovirus was included as an outgroup. Statistical supports of 100,000 ultrafast bootstraps are indicated at the nodes. Bold indicates sequences generated in this study; INSDC or GenBank accession numbers are provided. Scale bar indicates nucleotide substitutions per site.

De novo assembly of reads related to *Picornaviridae* generated a complete genome of a novel virus, tentatively named ovine picornavirus (OvPV). The obtained 7.5 kb OvPV genome is only very distantly related to known picornaviruses, with nucleotide sequence identities of 59% with a bovine picornavirus (International Nucleotide Sequence Data Collaboration [INSDC] accession no. LC006971) (*3*) and 55% with a canine picornavirus (accession no. KU871312) (*4*). The OvPV genome comprises a 5′ UTR, a predicted 6,885-nt open reading frame encoding the polyprotein with 2,294 aa, a 3′ UTR, and a poly(A)-tail. The putative structure of the polyprotein is similar to sapeloviruses with the characteristic order 5′-L-1ABCD-2ABC-3ABCD-3′; 3 consecutive methionins in frame could act as start codons of the polyprotein. The translation initiation site is proposed to be the third, because it is contained in the best Kozak context (*5*). Pairwise amino acid identities with P1, P2, and P3 of related picornaviruses are <58% (Appendix Table). Within the family *Picornaviridae,* together with other unclassified viruses (*4*,*6*), OvPV forms a putative new genus that is closest related to the genera *Sapelovirus*, *Rabovirus*, and *Enterovirus* (Figure 2; Appendix Figure). All whole-genome sequences we identified are available from the INSDC databases (accession no. PRJEB28719).

We attempted virus propagation from selected samples of affected animals on various mammalian cell lines (Appendix); however, virus could not be isolated. This result is consistent with other described unsuccessful attempts of viral propagation of related picornaviruses in cell culture (*4*,*7*).

To investigate whether the newly detected virus emerged in 2016 or was present previously, we examined archived cases from sheep in England with nonsuppurative encephalomyelitis oriented on gray matter that had tested negative for Louping ill virus by immunohistochemistry. Although the impaired RNA extracted from formalin-fixed paraffin-embedded (FFPE) material proved to be challenging for sequencing and amplification, we identified several additional cases of OvPV infection using reverse transcription PCR, and we generated whole-genome sequences from selected cases (Table 2). We detected OvPV in lambs of different breeds <3–4 weeks of age with neurologic signs; the earliest case identified dated back to 1998 (Table 2). Compared with the 2017 cases in Wales, the full OvPV genomes of the archive cases had ≈90%–96% sequence identity at the nucleotide level but up to 99% sequence identity at the amino acid level.

**Table 2:** Clinical history of lambs in England with nonsuppurative encephalomyelitis and results of testing of archived samples for presence of OvPV in spinal cord and brain*

Year	Signalment (case reference)	Case information	RT-PCR†	OvPV reads, no. (%)‡	Reference coverage§	Nucl. seq. identity§
1998	3-week-old mule lamb (1454/98)	Clinical signs of head tilt, rolling eyes and star gazing, and rapid onset of ataxia, recumbency, and paddling; no response to antimicrobial drugs, multivitamins including B1. Negative for Louping ill virus antibodies in cerebrospinal fluid and serum.	Positive	570 (0.003)	92%	90%–96%
2000	2–4-week-old milking breed lamb (960/00)	One of ≈12 lambs fed artificial colostrum (snatched at birth to prevent transmission of maedi-visna virus) that developed neurologic signs at ≈2 weeks of age, first weak in forelimbs, then hindlimbs, and by the second day, tremor and incoordination.	C_q_ 35	1061 (0.01)	98%	95%–96%
2004	Two 7–8-day-old triplet lambs (E1028/04 and E1029/04)	Triplets fed proprietary powdered colostrum because the ewe had insufficient milk. Signs of fine tremor progressing to recumbency; the third triplet developed similar neurologic signs at 3 weeks of age (not submitted for investigation).	C_q_ 32; positive	747,777 (3.02); 238,627 (1.11)	Full; full	95%; 95%
2008	2-week-old Texel X Swaledale (S313-04-08-1)	Neurologic signs not otherwise specified.	C_q_ 36	242 (0.0006)	89%	93%–95%
2010	1-week-old mule X female lamb (S78-04-10-1)	Approximately 25 lambs have been similarly affected. Portions of fixed juvenile ovine brain were submitted from field postmortem examination with history of suspected neurologic disease before euthanasia.	C_q_ 24	18,572 (0.07)	Full	95%
2014	Breed and age not known (S247-04-14)	Preweaned lamb with neurologic signs. Field postmortem samples submitted with clinical history of “suspect swayback,” thus likely clinical signs of ataxia and paresis.	C_q_ 34	NP	NP	NP

*C_q_, PCR quantification cycle; NP, not performed; nucl. seq., nucleotide sequence; OvPV, ovine picornavirus; RT-PCR, reverse transcription PCR; qRT-PCR, quantitative RT-PCR.  †RT-PCR was performed as prescreening for sample selection for high-throughput sequencing. Where feasible, C_q_ values of the qRT-PCR are indicated; positive means positive in semi-nested PCR. ‡Number (proportion) of OvPV reads in the complete high-throughput sequencing dataset. §Genome coverage and nucleotide sequence identity of the full genome or several contigs in relation to the OvPV reference genome strain OvPV/Wales/2017/S014175 (International Nucleotide Sequence Data Collaboration accession no. LR216008).

We performed in situ hybridization on OvPV PCR-positive FFPE tissues, with a subset of neurons within the brain and spinal cord as hybridization targets (Figure 1). In the spinal cord, OvPV in situ hybridization–positive neuronal soma and their projections were dense in the ventral horn in regions of neuronal degeneration and gliosis (Figure 1, panel C). Where dorsal root ganglia were in section, probe hybridization was detected in individual neurons (Figure 1, panels D, G). No hybridization was detected in sequential sections of OvPV-positive tissues under probing with an unrelated probe (Figure 1, panel E), and matched sections of uninfected animals were consistently negative (not shown).

Complete clinical data were not available for all of the archived cases; however, information was available for 5 flocks. In 4 of these flocks, the affected lambs were being fed artificially, usually because of maternal death or insufficient colostrum. Therefore, insufficient colostral antibody intake may predispose to development of neurologic disease similar to that proposed for porcine teschovirus, but this remains unproven. On the basis of the finding that OvPV has already been present in Wales, Scotland, and England for >20 years, we suspect that OvPV could be present in the intestinal tract commonly, similar to porcine teschovirus and porcine sapelovirus (*8*,*9*), but only occasionally causing disease in neonatal and early juvenile lambs.

## Conclusions

In this study, we report identification of a novel neuroinvasive picornavirus associated with severe nonsuppurative encephalomyelitis and sensory ganglionitis in sheep. The virus has been associated with encephalomyelitis for >20 years, affecting sheep in Scotland, Wales, and England in a range of sheep breeds and management systems, but solely young lambs to date. These findings could suggest that OvPV infection is usually subclinical and self-limiting, particularly in older animals. For some cases, particularly those involving multiple affected lambs in a single flock over 1 lambing season, the clinical histories of snatching at birth or artificial feeding of orphan or pet lambs indicated likely failure of transfer of colostral antibodies. The resulting colostrum deficiency may have acted as a predisposing factor. Further research is needed to determine geographic distribution, disease burden, transmission route, and other factors; a serologic assay is also needed to further analyze the distribution in the field. However, OvPV should be considered in the differential diagnosis of ovine nonsuppurative encephalomyelitis oriented on gray matter, particularly in young lambs with sensory ganglionitis.

Appendix Discussion of the materials and methods in study of picornavirus in lambs with severe encephalomyelitis.

## References

[1] Watson PJ, Scholes SF. Polioencephalomyelitis of unknown aetiology in a heifer. Vet Rec. 2004;154:766–7. 15224599

[2] Scheuch M, Höper D, Beer M. RIEMS: a software pipeline for sensitive and comprehensive taxonomic classification of reads from metagenomics datasets. BMC Bioinformatics. 2015;16:69. 10.1186/s12859-015-0503-6 25886935 PMC4351923

[3] Nagai M, Omatsu T, Aoki H, Kaku Y, Belsham GJ, Haga K, et al. Identification and complete genome analysis of a novel bovine picornavirus in Japan. Virus Res. 2015;210:205–12. 10.1016/j.virusres.2015.08.001 26260333 PMC7114519

[4] Woo PCY, Lau SKP, Choi GKY, Huang Y, Sivakumar S, Tsoi HW, et al. Molecular epidemiology of canine picornavirus in Hong Kong and Dubai and proposal of a novel genus in Picornaviridae. Infect Genet Evol. 2016;41:191–200. 10.1016/j.meegid.2016.03.033 27051044

[5] Kozak M. An analysis of 5′-noncoding sequences from 699 vertebrate messenger RNAs. Nucleic Acids Res. 1987;15:8125–48. 10.1093/nar/15.20.8125 3313277 PMC306349

[6] Pankovics P, Boros A, Reuter G. Novel picornavirus in domesticated common quail (*Coturnix coturnix*) in Hungary. Arch Virol. 2012;157:525–30. 10.1007/s00705-011-1192-8 22170478

[7] Lau SK, Woo PC, Yip CC, Choi GK, Wu Y, Bai R, et al. Identification of a novel feline picornavirus from the domestic cat. J Virol. 2012;86:395–405. 10.1128/JVI.06253-11 22031936 PMC3255865

[8] Chiu SC, Hu SC, Chang CC, Chang CY, Huang CC, Pang VF, et al. The role of porcine teschovirus in causing diseases in endemically infected pigs. Vet Microbiol. 2012;161:88–95. 22889731 10.1016/j.vetmic.2012.07.031

[9] Schock A, Gurrala R, Fuller H, Foyle L, Dauber M, Martelli F, et al. Investigation into an outbreak of encephalomyelitis caused by a neuroinvasive porcine sapelovirus in the United Kingdom. Vet Microbiol. 2014;172:381–9. 24984944 10.1016/j.vetmic.2014.06.001

