# Supplementary material for: Novel Picornavirus in Lambs with Severe Encephalomyelitis
Source: Emerg Infect Dis. 2019 May;25(5):963–7. doi: 10.3201/eid2505.181573 (PMC6478204; doi:10.3201/eid2505.181573)
Supplement: Appendix — Discussion of the materials and methods in study of picornavirus in lambs with severe encephalomyelitis. [file 18-1573-Techapp-s1.pdf]

Article DOI: https://doi.org/10.3201/eid2505.181573

# Novel Picornavirus in Lambs with Severe Encephalomyelitis

## Appendix

## Materials and Methods

### RNA Extraction

We disrupted the raw sample material using cryoPREP (Covaris, https://covaris.com) with subsequent lysis in 1 mL Buffer AL (QIAGEN, https://www.qiagen.com) and extracted RNA using Trizol LS Reagent (Thermo Fisher Scientific, https://www.thermofisher.com) in combination with RNeasy Mini Kit (QIAGEN) including DNase digestion (QIAGEN) on the spin column. We extracted formalin-fixed paraffin-embedded (FFPE) samples using the miRNeasy FFPE Kit (QIAGEN) as described by the manufacturer.

### Library Preparation and Sequencing

For metagenomic analysis, we processed samples as previously described (*1*) with a few adaptations of the protocol. We reverse transcribed RNA using the cDNA Synthesis System Kit (Sigma-Aldrich, https://www.sigmaaldrich.com) together with Hexanucleotide Mix (Sigma-Aldrich). We performed fragmentation on a M220 focused ultrasonicator (Sigma-Aldrich) for a final library size of 550 bp for native sample material and ≈200 bp for FFPE material. Subsequently, we prepared libraries using the GeneRead DNA Library I Core Kit (QIAGEN) according to the manufacturer's instructions, with the exception of using custom Y-adapters feasible for sequencing on Ion Torrent platforms. We performed size exclusion for the libraries prepared from native material (cases S012722-1 (lib01970-71), S012722-2 (lib01972-73), S014175 (lib02262) and S014177 (lib02263) as described previously (*1*). However, we purified libraries prepared from FFPE material (cases 1454/98 , 960/00, E1028/04, E1029/04, S313-04-08-1 and S78-04-10-1, corresponding to lib02670-75) only with 1.8x volume Agencourt AMPure XP Beads (Beckman Coulter, https://www.beckmancoulter.com), amplified them in 8 PCR cycles using the GeneRead DNA L Amp Kit (QIAGEN) according to the manufacturer's

instructions, and afterward purified them 2 times with 1.2x volume Agencourt Ampure XP Beads. After performing quality control on a High Sensitivity-Chip (Agilent Technologies, https://www.agilent.com), we quantified all libraries using KAPA Library Quantification Kit (Roche, https://www.kapabiosystems.com/) and sequenced them on an Ion Torrent platform (Ion Torrent PGM or Ion S5 XL, Thermo Fisher Scientific).

### Data Analysis

We classified the resulting raw reads using the metagenomic software pipeline RIEMS. We extracted reads belonging to the family *Picornaviridae* and assembled them de novo using the 454 Newbler software v2.6 and v3.0 (Roche). Thereafter, we mapped the whole dataset using Newbler v3.0 against the assembled full genome sequence for validation of the sequence, with a mean sequencing depth of 45 and 20 for the two OvPV genome sequences generated in the native spinal cord material. We assembled OvPV genomes of archived samples in an iterative mapping and assembly approach, with a mean sequencing depth of 340 to several thousand. We used EMBOSS Needle Pairwise Alignment v6.3.1 (https://www.ebi.ac.uk) to determine pairwise full genome sequence identities, as well as pairwise amino acid sequence identities for P1, P2, P3, and $3D^{pol}$. For phylogenetic analysis of full genomes, we aligned sequences using MAFFT v7.308 (https://mafft.cbrc.jp), followed by phylogenetic analysis with IQ-TREE v1.6.5 including search for the best-fit model with ModelFinder and 100,000 ultrafast bootstraps for statistical support. We constructed the phylogenetic tree using representative complete coding sequences of each species belonging to Sapeloviruses and Enteroviruses, based on the ICTV Master Species List 2016 v1.3 or, if not yet classified, the National Center for Biotechnology Information reference sequences. For phylogenetic analysis of peptide sequences, we aligned sequences using ClustalW (https://www.genome.jp/tools-bin/clustalw) with the BLOSUM scoring matrix, again followed by phylogenetic analysis with IQ-TREE v1.6.5 including search for the best-fit model with ModelFinder and 100,000 ultrafast bootstraps for statistical support.

### Real-Time Reverse Transcription-PCR (RT-qPCR)

The designed RT-qPCR amplifies a 110–bp-long target in the 5′-UTR region. The sequences of primers and probes are as follows: OvPV-215-F (5′-TGAGATGAGGGTTCAGTGGC-3), OvPV-319-R (5′-TAGCACACTCGTGGCTTCAG-3′), OvPV-249-FAM (5′-FAM-TAGACTGATCCCTGCGCTGGCTCAC-BHQ1–3′). We performed

the RT-qPCR using the QuantiTect Probe RT-PCR Kit (QIAGEN) in a 12.5 μL scale with 5 μM primers, 1.25 μM probe, and 2.5 μL sample. The thermal profile was as suggested by the manufacturer, with 50°C for 30 min, followed by 95°C for 15 min, and 45 cycles of 94°C for 15 s and 60°C for 60 s. We applied a primer-probe-system detecting β-actin as an internal control (*2*). In case of the strongly degraded RNA extracted from archived FFPE samples, we performed an additional PCR (semi-nested) amplifying an 80 bp product. For the semi-nested PCR, we used the OvPV forward primer in combination with OvPV-319-R 5′-TAGATTCAGTGCACGAAGCC-3′.

#### Viral Culture

We obtained the following cultured cells from the Collection of Cell Lines in Veterinary Medicine (CCLV) at the Federal Research Institute for Animal Health, Insel Riems, Germany: CCLV RIE0043 (SFT-R), CCLV RIE0194 (BHK-21 [BSR/5]), CCLV RIE0164 (BHK-21 [CT]), CCLV RIE0228 (Vero), CCLV RIE0132 (Neuro-2a) and CCLV RIE0127 (ZZ-R). For inoculation, we homogenized sample material (brain and spinal cord) in 1 mL of minimum essential medium (MEM) using the TissueLyser (QIAGEN) at 3000 Hz for 1–2 min. After centrifuging them for 5 min at 10,000 rpm, we inoculated different volumes (100 μL, 50 μL, and 10 μL) of the supernatant onto cells in a 24-well plate format. We incubated the cells at 37°C in a 2.5% $CO_2$ atmosphere and checked for cytopathic effects once a day. After 3–6 days, depending on the viability of the cells, we performed a freeze-thaw-cycle and transferred 100 μL of the culture lysate to a new passage of cells. In total, we conducted 3 blind passages and tested for viral replication using the established RT-qPCR.

#### In Situ Hybridization (ISH)

We performed colorimetric ISH manually on 5 μm sections of FFPE tissue on Superfrost Plus slides (Fisher Scientific, https://www.fishersci.com) using the RNAscope 2.5 Red assay kit (Cat #322360, Advanced Cell Diagnostics, Inc., https://acdbio.com). We designed V-Picornavirus-O1, ACD Cat #555011, as 35ZZ paired probe sets targeting region 967–3210 of the viral genome (INSDC accession no. LR216011). We pretreated each 5 μm section of FFPE tissue with heat and protease before probe hybridization for 2 h at 40°C. Negative controls used for validation of signal included an unrelated (GC-content matched) probe and an uninfected animal. We performed negative controls on serial sections. We counterstained slides with

hematoxylin and mounted them with EcoMount (Biocare Medical, https://biocare.net). We digitized slides using an Olympus VS120 scanner (https://www.olympus-lifescience.com) and a 40× objective with brightfield illumination.

## References


1. Wylezich C, Papa A, Beer M, Höper D. A versatile sample processing workflow for metagenomic pathogen detection. Sci Rep. 2018;8:13108. http://dx.doi.org/10.1038/s41598-018-31496-1

2. Toussaint JF, Sailleau C, Breard E, Zientara S, De Clercq K. Bluetongue virus detection by two real-time RT-qPCRs targeting two different genomic segments. J Virol Methods. 2007;140:115–23. http://dx.doi.org/10.1016/j.jviromet.2006.11.007


**Appendix Table.** Pairwise amino acid identities and similarities of P1, P2, P3 and $3D^{pol}$ of several unclassified species and representatives of the genera *Rabovirus*, *Sapelovirus*, and *Enterovirus* with the novel ovine picornavirus (strain OvPV/Wales/2017/S014175, INSDC accession no. LR216008).

| Genus | Name | INSDC accession no. | Pairwise amino acid identities with OvPV (%) | | | | Pairwise amino acid similarities with OvPV (%) | | | |
|---|---|---|---|---|---|---|---|---|---|---|
| | | | P1 | P2 | P3 | $3D^{pol}$ | P1 | P2 | P3 | $3D^{pol}$ |
| Unclassified | Bovine picornavirus | BAS29596 | 57.9 | 44.3 | 55.8 | 60.4 | 72.1 | 63.0 | 72.2 | 75.4 |
| | Feline picornavirus | YP_004934011 | 49.1 | 39.2 | 48.4 | 56.7 | 68.5 | 56.7 | 65.7 | 74.1 |
| | Canine picornavirus | YP_005351240 | 49.6 | 38.0 | 53.5 | 61.2 | 68.2 | 54.0 | 70.2 | 77.3 |
| | Ia io picornavirus 1 | AFK85008 | 47.8 | 38.7 | 50.7 | 57.6 | 64.8 | 54.7 | 68.2 | 74.6 |
| *Rabovirus* | Rabovirus A | YP_009118268 | 39.9 | 38.1 | 45.8 | 54.0 | 57.4 | 55.4 | 62.0 | 72.2 |
| *Sapelovirus* | Avian Sapelovirus | YP_164335 | 41.2 | 26.3 | 45.6 | 54.0 | 57.2 | 36.7 | 63.2 | 69.9 |
| | Sapelovirus A | NP_653145 | 41.4 | 31.3 | 47.2 | 55.3 | 58.6 | 45.1 | 63.8 | 69.9 |
| | Sapelovirus B | NP_758809 | 38.2 | 31.3 | 50.1 | 57.2 | 53.2 | 45.1 | 66.8 | 70.7 |
| *Enterovirus* | Enterovirus A | NP_042242 | 36.9 | 28.4 | 45.3 | 50.2 | 52.3 | 44.2 | 63.6 | 67.5 |
| | Rhinovirus A | NP_042288 | 36.3 | 31.7 | 46.1 | 52.7 | 52.0 | 46.4 | 63.6 | 70.7 |
| | Rhinovirus B | NP_041009 | 35.0 | 28.7 | 45.4 | 52.3 | 51.8 | 47.2 | 65.0 | 71.5 |
| | Enterovirus C | NP_041277 | 34.6 | 29.3 | 44.3 | 50.9 | 51.0 | 45.5 | 64.8 | 69.4 |
| | Rhinovirus C | YP_001552411 | 35.8 | 32.0 | 43.7 | 49.8 | 50.8 | 46.3 | 62.1 | 68.9 |
| | Enterovirus B | NP_040958 | 35.2 | 28.5 | 45.3 | 51.1 | 50.6 | 45.5 | 64.6 | 69.3 |

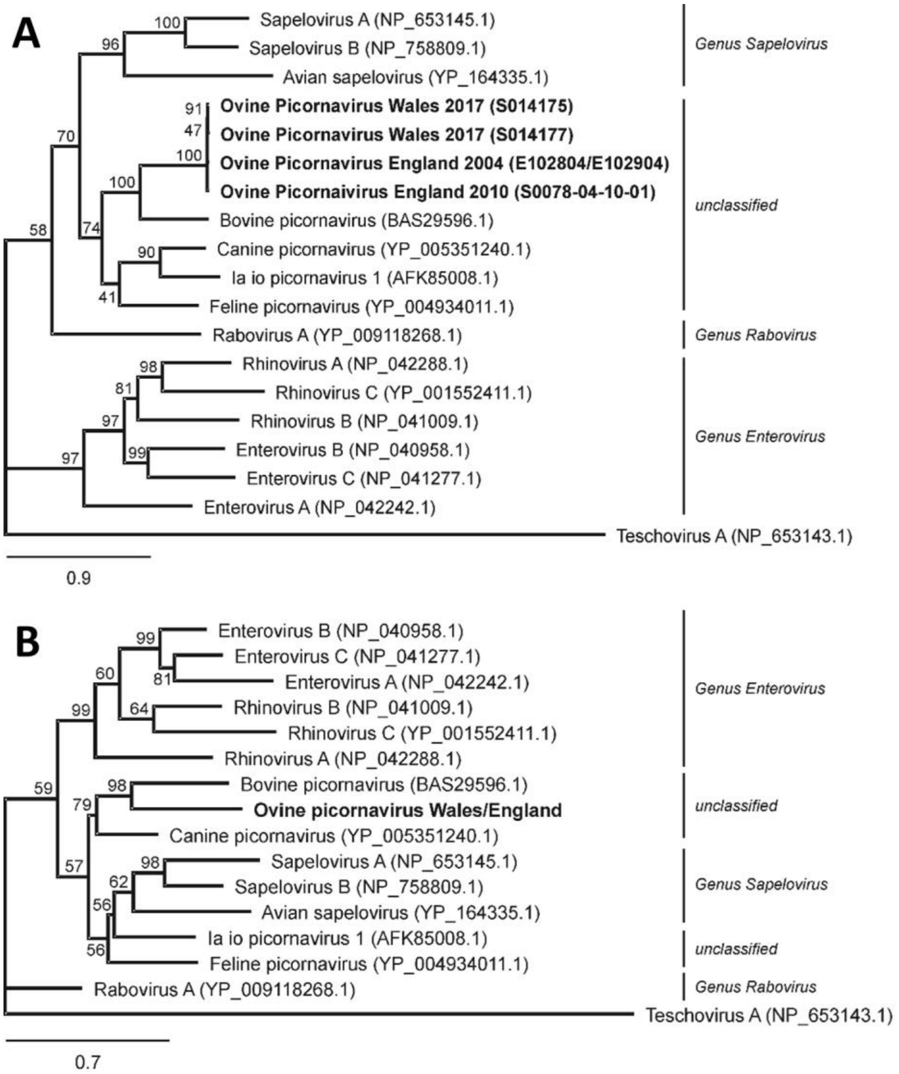


**Appendix Figure.** Phylogenetic analysis of the amino acid sequences of A) P1 and B) 3D$^{pol}$ of related picornavirus genera and unclassified species. The 3D$^{pol}$ amino acid sequences of the full OvPV genomes generated in this study are identical. The maximum-likelihood phylogenetic trees were calculated by IQ-TREE v1.6.5 with the best-fit model LG+F+I+G4 for P1 and LG+G4 for 3D$^{pol}$. Teschovirus A was included as an outgroup. Statistical support of 100,000 ultrafast bootstraps are indicated at the nodes. Scale bar represents nucleotide substitutions per site.
